# Trephining for Cerebral Tumour

**Published:** 1890-01-18

**Authors:** 


### TREPHINING FOR CEREBRAL TUMOUR.

We have already referred to several recent cases of brain surgery, all tending to show the great advances which have been made in this department of the science during recent years. There is now reported a case of cerebral tumour successfully removed by trephining, by Mr. Rushton Parker, professor of surgery in University College, Liverpool. The symptoms were long-standing headaches, mental dulness, paralysis of left arm, weakness of left leg. Iodide of potassium for three months did not relieve the symptoms. A puffy swelling now appeared in front of the right parietal eminence. What this might have to do with the supposed tumour inside was unknown. But as it was in the region which the symptoms would lead to choose, it was accepted as the guide, and found to consist of thickened and softened pericranium. On trephining with a 1½in. instrument a rounded tumour, the size of a walnut, with a bit of adherent dura mater, was shelled out by breaking through the surrounding brain with the fingers. On the third day, in consequence of free discharge of serum, drainage tubes were introduced. But then followed "pulsation over the hole, and suppuration in their track," with return of paralysis. On the nineteenth day the almost healed flap was turned down again. The brain being œdematous and prominent was sliced off level with the bone, revealing sinuses lined with puriform lymph, which was scraped away and the wound packed. No more bad symptoms occurred, excepting a large fungus which was eventually got rid of. Gradual improvement continued, and at the end of five months the patient could turn over the leaves of a book. There was total loss of headache and of the previous dazed feeling, and he expressed himself as restored to mental vigour and comfort. Mr. Rushton Parker considers the case was one of gumma, as it was ascertained the patient had been treated for a periosteal swelling of the sternum six years previously. It would be very interesting to ascertain the future of this patient. Many cases, the future history of which would be of great scientific value, become lost when removed from the immediate cognisance of the medical attendant; and it certainly would be well if some means were devised by which the history of such cases could be preserved. This does not appear impossible to accomplish, at least as regards many patients.

